# Multi-domain cognitive impairments at school age in very preterm-born children compared to term-born peers

**DOI:** 10.1186/s12887-021-02641-z

**Published:** 2021-04-13

**Authors:** Elise Roze, Sijmen A. Reijneveld, Roy E. Stewart, Arend F. Bos

**Affiliations:** 1 Division of Neonatology, Beatrix Children’s Hospital, University Medical Center Groningen, University of Groningen, Groningen, Netherlands; 2 Divison of Neonatology, Wilhelmina Children’s Hospital, University Medical Center Utrecht, Utrecht, Netherlands; 3 Department of Health Sciences, University Medical Center Groningen, University of Groningen, Groningen, Netherlands

**Keywords:** Prematurity, Long-term outcome, Co-occurrence, Cognition, Neurodevelopmental outcome

## Abstract

**Background:**

Preterm infants are at risk for functional impairments in motor, cognitive, and behavioral development that may persist into childhood. The aim of this study was to determine the co-occurrence of cognitive impairments in multiple cognitive domains at school age in very preterm born children compared to term-born children.

**Methods:**

Comparative study including 60 very preterm-born children (gestational age ≤ 32 weeks) and 120 term-born controls. At school age, we assessed intelligence with the WISC-III, and visuomotor integration with the NEPSY-II, verbal memory with the AVLT, attention with the TEA-ch, and executive functioning with the BRIEF. We investigated co-occurrence of various abnormal (<5th percentile) and suspect-abnormal (<15th percentile, including both suspect and abnormal) cognitive functions.

**Results:**

At mean age 8.8 years, 15% of preterm children had abnormal outcomes in multiple cognitive functions (≥2), versus 3% of the controls (odds ratio, OR 4.65, 95%-confidence interval, CI 1.33–16.35). For multiple suspect-abnormal cognitive outcomes, rates were 55% versus 25% (OR 3.02, 95%-CI 1.49–6.12). We found no pattern of co-occurrence of cognitive impairments among preterm children that deviated from term-born controls. However, low performance IQ was more frequently accompanied by additional cognitive impairments in preterms than in controls (OR 5.43, 95%-CI 1.75–16.81).

**Conclusions:**

A majority of preterm children showed co-occurrence of impairments in multiple cognitive domains, but with no specific pattern of impairments. The occurrence of multi-domain cognitive impairments is higher in preterms but this seems to reflect a general increase, not one with a pattern specific for preterm-born children.

**Supplementary Information:**

The online version contains supplementary material available at 10.1186/s12887-021-02641-z.

## Introduction

Preterm birth is a relative common complication of pregnancy. Very preterm birth (< 32 weeks gestation) occurs in around 1–2% of pregnancies and accounts for ~ 2.4 million births per year worldwide [1]. In the early neonatal period, various factors from the extra-uterine environment contribute to the risk of mild, to occasionally more severe brain lesions in preterm infants. Early disruptions and alterations in brain development can lead to functional impairments in motor, cognitive, and behavioral development that may persist into childhood and even adulthood [2, 3]. These functional impairments are likely to have an impact on daily life and academic achievement [4, 5].

The cognitive impairments found in preterm children are not restricted to a poorer intellectual development leading to lower intelligence. Other cognitive functions like attention, visuomotor integration, and executive functioning can also be affected [6, 7]. It is unclear whether a small subset of children are responsible for the lower means on all of these cognitive functions in preterm children while others score in the average range, or whether the majority of preterm children have some cognitive impairments in at least one cognitive domain.

Previous studies reported that specifically preterm children with low intelligence are at risk for problems in other cognitive domains [8, 9], while others suggested there may be preterm children with specific cognitive dysfunctions in whom intellectual development is preserved [10, 11]. In addition, it has been suggested that deficits in specific cognitive domains, such as processing speed, mediate deficits in other cognitive domains, such as executive functioning [12].

However, very few studies described the co-occurrence of impairments in cognitive functions in preterm children. One study described that intellectual and learning disabilities are associated with poor executive function, visuo-spatial and sensori-motor skills [13]. Other studies linked impairments in executive functions and visuo-spatial processing to poorer academic achievement [14–16].

Insight into co-occurrence of cognitive impairments in preterm children could.

reveal whether (i) high risk preterm children develop a pattern of co-occurring impairments in specific cognitive functions, or (ii) multiple cognitive functions are impaired in preterm children without a pattern emerging, or (iii) in most children a single cognitive function is impaired. Insight into patterns of cognitive impairments could improve understanding of underlying pathophysiological mechanisms and could guide diagnostics and intervention. Co-occurrence of cognitive impairments may point to specific brain areas involved in these functions that are particularly vulnerable for preterm birth. In addition, if for example children with inattentiveness also have problems in executive functioning, they could be screened more specifically for these functions at an early age, to start training and appropriate guidance as early as possible.

Our primary aim was to investigate the co-occurrence of cognitive impairments at school age in a Dutch cohort of very preterm-born children (gestational age ≤ 32 weeks), and to compare them with term-born children. Our second aim was to determine if certain cognitive impairments more frequently co-occur with other cognitive impairments and whether this pattern of cognitive impairments relates to educational achievement.

## Methods

### Participants

This comparative study included 60 randomly selected preterm-born infants (gestational age ≤ 32 weeks) who had been admitted to the neonatal intensive care unit (1996–2002). Most of these children had participated in previous cohort studies on the outcome of preterm children with specific neonatal risk factors, either as a case or control. We did not include all preterm children that participated in previous cohort studies since certain neonatal risk factors would be over represented. We randomly selected the number of cases with a specific neonatal risk factor based on the incidence of the risk factor in 1996–2002. We excluded children with major chromosomal and congenital anomalies. The response rate at school age was 85%. For every preterm child, we included two randomly selected term-born control children to increase the power of the study [17]. Controls were born in 2002 and 2003 and are a random community-based sample from 13 preventive child health centers in the Netherlands. They were initially recruited for the Lollipop study as described elsewhere [18]. Of the term born controls the response rate at school age was 73%.

### Measures and procedure

We reviewed the preterm-born children’s medical charts for neonatal characteristics.

For patient demographics, see the supplementary Table 1.

Socioeconomic status (SES) was classified according to the highest occupation of both parents into below average, average, and above average according to the international standard classification of occupations [19]. The preterm-born children and term-born controls were invited prospectively to participate in an extension of our routine follow-up program. It entailed the assessment of motor performance, cognitive functions, and behavior at the age of 6–12 years. Including breaks the program took approximately 2.5 h to complete. We report here on the children’s cognitive functions, as we were particularly interested in their cognitive development.

### Cognitive measures

Total, verbal, and performance intelligence were assessed using a shortened version of the Wechsler Intelligence Scale for Children, third edition, Dutch version (WISC-III-NL). This shortened screening version of the WISC-III correlates quite well with the total assessment [20].

In addition, we assessed visuomotor integration with the subtest Design Copying of the NEPSY-II, a neuropsychological test battery for children [21]. In this subtest the child is asked to copy geometric shapes of increasing complexity. Visuomotor integration involves the integration of visual information with finger-hand movements.

We assessed verbal memory with a standardized Dutch version of Rey’s Auditory Verbal Learning Test [22]. This test consists of five learning trials of fifteen words with immediate recall after each presentation and a delayed recall trial followed by a recognition trial.

We measured selective attention and attentional control with the subtests Map Mission and Opposite Worlds of the Test of Everyday Attention for Children, Dutch version (TEA-Ch-NL) [23]. Selective attention refers to a child’s ability to select target information from an array of distractors. Attentional control refers to the child’s ability to shift attention flexibly and adaptively. Finally, the parents filled out the Behavior Rating Inventory of Executive Function to assess executive functioning involved in well-organized, purposeful, goal-directed, and problem-solving behavior as observed by the parents in daily life [24]. If children were too tired and/or uncooperative (as assessed by the trained experimenter), we excluded their test scores. In addition, we recorded the type of education the children received and whether they had repeated classes. Cognitive testing was performed by a neuropsychologist, or a trained test-assistant under direct supervision of a neuropsychologist.

### Statistical analyses

We used age-specific norm scores as provided in the manuals for all the cognitive tests. We classified the intelligence quotients (IQs) into normal (IQ ≥ 85), suspect (IQ 70 to 85) and abnormal (IQ < 70). We used the percentiles on the standardization samples of the cognitive tests to classify raw scores into normal (≥ 15th percentile), suspect (5th to 15th percentile), and abnormal (< 5th percentile). Verbal memory and attention have two different parameters that each measure different aspects of these functions. For each child we classified verbal memory and attention as suspect or abnormal if either one of the two parameters was suspect or abnormal, always taking the poorest outcome. To compare the cognitive test scores of preterm children and controls we used the Student’s *t*-test and the Mann-Whitney U test.

Second, we explored patterns of co-occurrence of cognitive impairments by graphically displaying the co-occurrence of abnormal (< 5th percentile) and suspect-abnormal (< 15th percentile, including both suspect and abnormal) outcomes of each child. We grouped children with suspect and abnormal scores together since they may all be regarded as outside the normal range and therefore having a certain amount of impairment.

Next, to assess the degree of clustering of adverse outcomes, we compared the frequencies of combinations of impairments (i.e., no impairment, 1 impairment, 2 impairments, etc.) with those expected based on chance. The expected numbers were calculated based on the relative likelihood of each number of combinations derived from factorial combinations.

We assessed the statistical significance between the observed and expected distributions by chi-square tests, separately for preterms and controls. Effect sizes were expressed by Cohen’s W [25]. This analysis was corrected for age at follow-up to rule out any effect of age differences on the findings.

Third, we calculated odds ratios (ORs) for obtaining an abnormal outcome on at least one cognitive domain or multiple (≥2) abnormal cognitive domains in preterms versus controls by logistic regression analyses. These analyses were adjusted for gender and socioeconomic status. Since we used age-specific norm scores, we did not perform additional corrections for age at testing for this analysis.

Fourth, to gain insight into combinations of impairments, we tested differences in co-occurrence of cognitive impairments between preterms and controls using the chi-square test. We used SPSS 17.0 software for Windows (SPSS Inc., Chicago, IL) for all the analyses and *p* < .05 was considered statistically significant.

## Results

We included 60 preterm infants, that were 41 males and 19 females with median gestational age (IQR) 29.4 weeks (27.3–30.8), and birthweight 1138 g (941–1410)). Their SES was below average in 23%, average in 55% and above average in 22%. Of 120 control infants, 57 were male and 63 female, and SES was below average in 20%, average in 39% and above average in 41%. There was a preponderance of males in the preterm group compared to the controls (*p* < .01) and a trend towards lower socioeconomic status (*p* = .052), the latter being an expected finding. Mean age at follow-up was higher in the preterms (8.8 years, range 6.8y-12.8y) than in the controls (6.9 years, range 6.4y-7.1y, *p* < 0.05).

### Cognitive outcome

**Fig. 1:**
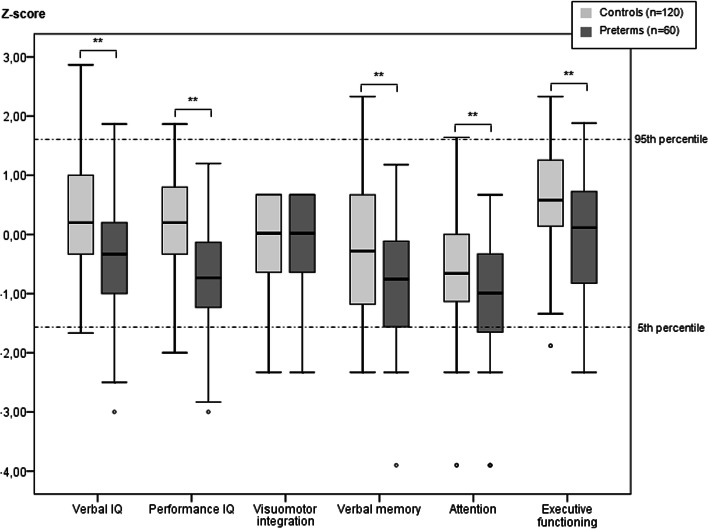
Standardized test scores of cognitive tests at school age in the preterm group (*n =* 60) compared to the control group (*n =* 120). Box-plots represent z-score distribution (median, 25th and 75th percentile and range).**p < 0.01

Mean total IQ in the preterm group was 92 (range 55–118), verbal IQ 95 (55–128), and performance IQ 90 (55–118). In the controls mean total IQ was 104 (76–132), verbal IQ 106 (75–143), and performance IQ 103 (70–128). The box-plots in Fig. 1 show the standardized test scores (z-scores) from the cognitive tests of the entire sample of preterm children and controls. Preterm children performed significantly poorer on all tests except on the test for visuomotor integration, which showed a ceiling effect in its distribution. The distributions of the cognitive test scores in the preterm group were fairly similar to those in the control group except that the means were lower in the former.

### Co-occurrence analyses

**Fig. 2:**
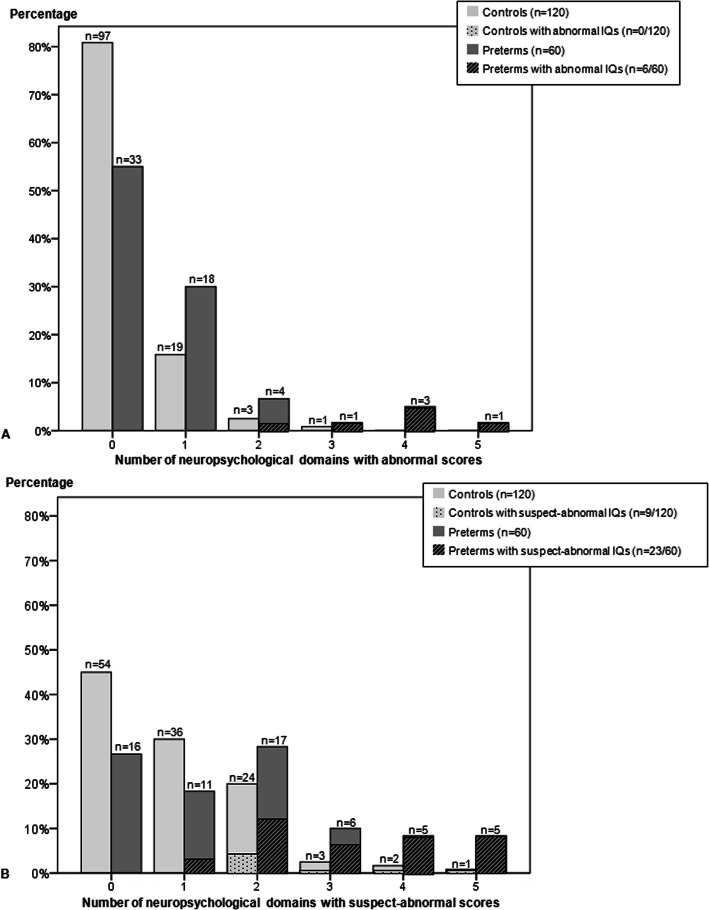
Percentages of children by number of either abnormal (**a**) or suspect-abnormal (**b**) cognitive outcomes of the six domains: verbal IQ, performance IQ, visuomotor integration, verbal memory, attention, and executive functioning. Suspect-abnormal IQs (< 85) or abnormal IQs (< 70) cover either verbal or performance IQ

Figure 2 shows the percentage of children per number of abnormal (A) or suspect-abnormal (B) cognitive domains. Out of 60 preterm children, 33 had no abnormal scores whilst 27 children (45%) had an abnormal result on at least one cognitive test. This was 23 out of 120 (19%) in the control group (OR 2.82, 95% confidence interval (CI) 1.39–5.37, *p* < .01). The number of preterm children with abnormal scores in multiple cognitive domains, i.e. in 2 or more, was higher than in the control group (9/60 (15%) versus 4/116 (3%), OR 4.65, 95% CI 1.33–16.35, *p* = .02, Fig. 2a).

When suspect and abnormal scores were taken together as an unfavorable result (Fig. 2b), then the majority of preterm children had unfavorable outcomes on multiple cognitive domains. This was again more frequent than in the controls (33/60 (55%) versus 30/120 (25%), OR 3.02, 95% CI 1.49–6.12, *p* < 0.01).

All preterm born children with an abnormal total IQ (< 70, Fig. 2a) had additional cognitive impairments in other domains. None of the control children had abnormal total IQs < 70.

### Patterns of co-occurrence

There were *n* = 18 preterm children with only one abnormal result (Table 1). Of these children, most had problems with verbal memory (*n* = 9), followed by visuomotor integration (*n* = 4), attention (*n =* 4), and executive functioning (*n =* 1). In the controls *n* = 19 had only one abnormal result (Table 1). Although this occurred less frequent in the control group, the distribution of impairments in cognitive functions was similar.

**Table 1:** Distributions of observed versus expected numbers of combinations of cognitive impairments in preterm and control children

	*Preterms*		*Controls*	
	*Observed*	*Expected*	*Observed*	*Expected*
None	33 (55%)	26.1 (43.6%)	97 (81%)	94.2 (78.5%)
One impairment^a^	18 (30%)	20.2 (33.7%)	19 (16%)	23.5 (19.6%)
Two impairments^a^	4 (7%)	11.6 (19.3%)	3 (3%)	2.2 (1.8%)^b^
Three impairments and over^a^	5 (8%)	2.0 (3.4%)	1 (1%)	0.1 (0.2%)^b^
*P*-value	0.01		0.35^b^	
Cohen’s W	0.43		0.13^b^	

^a^Refer to abnormal (< 5th percentile) test scores

^b^Two highest categories have been combined for statistical testing

Inspection of patterns of co-occurrence of cognitive impairments in the preterm children (*n* = 9) and controls (*n* = 4) with two or more abnormal scores revealed no clear pattern of combinations of abnormal outcomes (data not shown).

In Table 2, the frequency of suspect-abnormal outcomes in cognitive functions in children with one versus multiple (≥ 2) suspect-abnormal functions is shown. Within the group of preterm children with ≥2 suspect-abnormal outcomes, attention, verbal memory, and performance IQ were most affected.

**Table 2:** Type of cognitive impairment in children with one versus multiple cognitive impairments (≥ 2 suspect-abnormal domains)

	1 suspect-abnormal domain		≥2 suspect-abnormal domains	
	Preterms (*n* = 11)	Controls (*n* = 36)	Preterms (*n* = 33)	Controls *(n* = 30)
Verbal IQ	2 (18%)	0 (0%)	11 (33%)	5 (17%)
Performance IQ	0 (0%)	0 (0%)	19 (58%)**	6 (20%)
Visuomotor integration	0 (0%)	7 (19%)	14 (42%)	16 (53%)
Verbal memory	4 (36%)	14 (39%)	21 (63%)	23 (77%)
Attention	5 (45%)	15 (42%)	21 (63%)	15 (50%)
Executive functioning	0 (0%)	0 (0%)	11 (33%)	5 (17%)

Data are given as number of children with suspect-abnormal outcomes (percentage of the group concerned)

** *p* < 0.01 when comparing preterms with ≥2 suspect-abnormal domains to controls with ≥2 suspect-abnormal domains (Chi^2^)

Distributions of observed numbers of combinations of impairments differed from expected ones between preterms and term born children (Table 1). For preterms, high numbers of combinations of impairments occurred relatively more frequently than expected (*p* = 0.01). However for controls, the observed frequencies were near expected (*p* = .35). Clustering effects in preterms were rather large, Cohen’s effect size (Cohen’s W) being 0.43. After adjustment for age at follow-up, clustering of impairments did not occur more frequently in preterm-born children than in term-born children.

In comparison to the controls, a suspect-abnormal performance IQ was more frequently accompanied by additional cognitive impairments in preterm children than in control children (OR 5.43, 95% CI 1.75–16.81).

### Pattern of cognitive impairment in relation to type of education

Table 3 shows the type of education (normal education, normal education but repeated classes, special education) of the preterm and control children. It also provides the median number of suspect-abnormal cognitive domains per type of education. Out of the six preterm children that received special education, five attended special schools for children with learning problems and one a school for children with behavioral problems. In the control group, one child attended a school for children with learning problems and one a school for children with language and hearing problems. The median number of suspect-abnormal cognitive domains was significantly higher for children repeating classes and attending special education, both in the preterm (*p* < 0.01) and control groups (*p* < 0.01). In Table 4, the type of cognitive impairment in relation to type of education in preterm children is shown. Verbal IQ, performance IQ, visuomotor integration and attention were significantly more frequently affected in children repeating classes and children attending special education (*p* < 0.01).

**Table 3:** Number of impairments in cognitive domains in relation to educational achievement

Educational achievement	Preterm children (*n* = 60)		Control children (*n* = 120)	
Normal education	*n* = 31 (52%)	1 (0–2)	*n* = 112 (93%)	1 (0–1)
Normal education, repeated classes	*n* = 23 (38%)	3 (2–4)	*n* = 6 (5%)	2.5 (1.5–4)
Special educational classes	*n* = 6 (10%)	4.5 (1.5–5)	*n* = 2 (2%)	3.5 (2–5)

Data are given as number of children (percentage) followed by median number of cognitive domains with suspect-abnormal outcomes (25th–75th percentile)

**Table 4:** Type of impairment in cognitive domains in relation to educational achievement in the preterm group

	*Normal education (n = 31)*	*Normal education, repeated classes (n = 23)*	*Special educational classes (n = 6)*
Verbal IQ**	1 (3%)	8 (35%)	4 (67%)
Performance IQ**	2 (6%)	13 (57%)	4 (67%)
Visuomotor integration**	2 (6%)	7 (30%)	5 (83%)
Verbal memory	10 (32%)	11 (48%)	3 (50%)
Attention**	8 (26%)	15 (65%)	4 (67%)
Executive functioning	4 (13%)	6 (26%)	1 (17%)

Data are given as number of children with suspect-abnormal outcomes (percentage of the group concerned). ** *p* < 0.01 (Chi^2^)

## Discussion

This study showed that a majority of very preterm-born children had suspect-abnormal cognitive outcomes in multiple cognitive domains within a child. However, the degree of clustering of impairments in preterms was not greater than expected in this group. We could not identify any typical pattern of co-occurrence of impairments in specific cognitive domains among preterm children compared to controls. However, low performance IQ was more often associated with multiple cognitive impairments in preterm children than in controls. Multi domain cognitive impairments were related to type of education. Many preterm-born children thus had, presumably through altered brain development, impairments in combinations of cognitive domains that seemed to hamper educational achievement.

The novelty of this study is the co-occurrence of cognitive impairments in preterm children by reporting the number of affected cognitive domains and combinations of impairments rather than reporting average scores across preterm children. This provides insight into the effect of prematurity on later cognitive outcome. This analysis showed that although a majority of preterm children had problems in multiple cognitive domains, there was no specific clustering of impairments in preterms compared to term-born controls.

We could not identify a specific pattern of co-occurrence of cognitive impairments typical for preterm children, although most preterm children were impaired in multiple cognitive domains. Nevertheless, attention, verbal memory, and performance IQ were the cognitive domains most frequently involved in preterm children with suspect-abnormal outcomes in multiple cognitive domains. Previously, these functions have been identified as being affected by preterm birth [26, 27], but we found them also to be more commonly affected in combination with impairments in other cognitive domains when compared to term-born children.

Previous studies reported that additional cognitive impairments are found in preterm children with poor intellectual development [8, 13, 28]. E.g., a study of Johnson et al. found that intellectual disabilities were associated with problems in executive functions, visuo-spatial and sensori-motor skills. Their study did not report on attention and verbal memory, domains that were frequently affected in the present study. Others also stated that very preterm children can be at risk for cognitive impairment despite average intelligence [10, 11]. In our study, specifically low performance IQ was more frequently accompanied by additional cognitive impairments in the preterm group indicating that low performance IQ may be interpreted as a potential redflag of impaired neurodevelopment that poses a preterm born child at risk for impairments in other cognitive domains. However, a substantial group of preterm children had suspect-abnormal outcomes on domains in the absence of low IQs. Our study adds that in these children impairments are mostly confined to only one cognitive domain. Thus, including only general intellectual development in a follow-up program for preterm children may wrongfully classify a preterm child as developing normally, thereby missing possible additional cognitive problems.

In our study the number of suspect-abnormal cognitive domains was clearly related to educational achievement. The study by Johnson et al. also found that preterm children with intellectual disabilities had poorer neuropsychological abilities and curriculum-based attainment [13]. However in their study, increased special educational needs were found only in children with both intellectual and learning disabilities. In our study, not all children with special educational needs had intellectual impairment. Learning difficulties typical for preterm children, e.g. in the field of reading, spelling, mathematics, or writing can be independent of IQ scores [2, 3]. It may well be that combinations of problems in cognitive functions as found in the present study, such as attention and visuomotor integration, underlie such learning difficulties and special educational needs.

Several characteristics of preterm brain development may be involved in the underpinnings of these multi-domain cognitive impairments at school age. First, disruption of processes of brain connectivity such as synaptogenesis and corticogenesis that normally occur in the intra-uterine environment, can lead to structural changes in brain organization in preterm children that persist into young adulthood [29–31]. In addition to alterations in brain development, perinatal brain injury also affects brain maturation processes [32]. Particularly the white matter of preterm infants is vulnerable to damage, for example from inflammatory responses in case of sepsis [33]. These disruptions and alterations in brain development may have several functional implications. For example, diminished cortical volumes have been related to lower intellectual development [34], altered pathways of brain activation to attention allocation [35], and disturbed connectivity between posterior brain regions and the prefrontal cortex to poorer executive functions in preterm children [36]. Nevertheless, the direct functional implications of combinations of these processes, and how they lead to specific multi-domain cognitive impairments at a later age, remain poorly understood.

In our study, we found a lower SES in the preterm children compared to controls. Also, IQs were lower in the preterm group. SES has previously been related to both risk of preterm birth and lower intellectual outcome and therefore may act as a confounding factor. There have also been studies, i.e. by Richards et al. that suggest that SES also acts as a modifier of the effect of preterm birth on children’s cognitive development, in other words that lower SES exacerbated the adverse impacts of preterm birth [37]. These children deserve specific attention for cognitive impairments during follow-up.

This study was limited by a relatively small sample size. To increase the power of our study, we included twice as many controls as preterm children. Second, at follow-up, the control children were younger than the preterm children (6.9 vs 8.8 years). Our adjustment for age indeed shows that this may otherwise confound findings on the co-occurrence of impairments. Next, we had a slightly lower SES in the preterm group, but we could adjust for this in all comparisons. It is of note that executive function was assessed by parental report (which assesses day to day executive function), but not by a performance based executive function assessment. This was an exploratory study in which we chose to test those cognitive functions that have been reported to be poorer in preterm-born children. Due to limited attention span of the children, we were unable to test all possible functions, leaving out e.g. language functions. Cerebral pathology and other complications that occurred in about 25% of the children could also have contributed to the impairments.

## Conclusions

In conclusion, a majority of preterm children had suspect-abnormal outcomes on multiple cognitive domains. Preterms did not have more clustering of impairments than term-born children and no typical pattern of impairment in specific cognitive domains discriminates these two groups. The number of suspect-abnormal cognitive domains was related to poorer educational achievement. This study points to the necessity of early identification of children at risk for co-occurrence of cognitive impairment raising the question of how to implement intervention strategies that could contribute to improving the outcomes of these children. It is of importance to closely follow very preterm children at regular intervals and for multiple cognitive domains, including language, to timely detect impairments.

## Supplementary Information

**Additional file 1: Table S1.** Patient characteristics.

## Data Availability

The dataset analysed during the current study is available from the corresponding author on reasonable request.

## Abbreviations

BRIEF: Behavior Rating Inventory of Executive Function
CI: Confidence interval
IQ: Intelligence quotient
OR: Odds ratio
SES: Socioeconomic status
WISC: Wechsler Intelligence Scale for Children
