# Supplementary material for: Multi-domain cognitive impairments at school age in very preterm-born children compared to term-born peers
Source: BMC Pediatr. 2021 Apr 13;21:169. doi: 10.1186/s12887-021-02641-z (PMC8042721; doi:10.1186/s12887-021-02641-z)
Supplement: Supplementary file 1 — Additional file 1: Table S1. Patient characteristics. [file 12887_2021_2641_MOESM1_ESM.docx]

**Multi-domain cognitive impairments at school age in very preterm-born children compared to term-born peers**

Elise Roze, MD, PhD,^a,b^ Sijmen A. Reijneveld, MD, PhD,^c^ Roy E. Stewart, PhD,^c^ Arend F. Bos, MD, PhD^a^

^a^Division of Neonatology, Beatrix Children’s Hospital, University Medical Center Groningen, University of Groningen, Groningen, Netherlands

^b^Divison of Neonatology, Wilhelmina Children’s Hospital, University Medical Center Utrecht, Utrecht, Netherlands

^c^Department of Health Sciences, University Medical Center Groningen, University of Groningen, Groningen, Netherlands

**TABLE S1. Patient characteristics**

|  | Preterm infants (GA < 32 weeks) *n*=60 | Control infants *n*=120 |
| --- | --- | --- |
| Males/females | 41/ 19 | 57/63 |
| Gestational age (weeks) | 29.4 (27.3-30.8) | 40.0 (39.0-40.0) |
| Birth weight (grams) | 1138 (941-1410) | 3590 (3250-3945) |
| *Socioeconomic status** |  |  |
| Below average | 14 (23%) | 24 (20%) |
| Average | 33 (55%) | 47 (39%) |
| Above average | 13 (22%) | 49 (41%) |
| *Neonatal complications* |  |  |
| IUGR (<P10) | 11 (18%) |  |
| Apgar at 5 minutes | 9 (4-10) |  |
| Asphyxia | 1 (2%) |  |
| Ventilatory support (IPPV or HFO) | 50 (83%) |  |
| Early onset sepsis | 3 (5%) |  |
| *Cerebral pathology* |  |  |
| GMH-IVH grade I or II | 12 (20%) |  |
| GMH-IVH grade III or PVHI | 1 (2%) |  |
| PHVD | 2 (3%) |  |
| *Late onset morbidity* |  |  |
| Late onset sepsis | 24 (40%) |  |
| Necrotizing enterocolitis | 8 (13%) |  |
| Bronchopulmonary dysplasia | 20 (33%) |  |

Data are given as median (25th-75th percentile) or as numbers (percentage).

*According to the standard Dutch occupational classification

Abbreviations: GMH-IVH- germinal matrix hemorrhage-intraventricular hemorrhage; HFO- high frequency oscillation; IPPV- intermittent positive pressure ventilation; IUGR- intrauterine growth restriction; PHVD- post-hemorrhagic ventricular dilatation; PVHI- periventricular hemorrhagic infarction.
